# On the Significance of New Biochemical Markers for the Diagnosis of Premature Labour

**DOI:** 10.1155/2014/251451

**Published:** 2014-12-08

**Authors:** Rafał Rzepka, Barbara Dołęgowska, Aleksandra Rajewska, Sebastian Kwiatkowski

**Affiliations:** ^1^Department of Obstetrics and Gynecology, Pomeranian Medical University, 70-111 Szczecin, Poland; ^2^Department of Laboratory Diagnostics and Molecular Medicine, Pomeranian Medical University, 70-111 Szczecin, Poland

## Abstract

Preterm labour is defined as a birth taking place between 22nd and 37th weeks of gestation. Despite numerous studies on the aetiology and pathogenesis of preterm labour, its very cause still remains unclear. The importance of the cytokines and acute inflammation in preterm labour aetiology is nowadays well-proven. However, chronic inflammation as an element of the pathogenesis of premature labour is still unclear. This paper presents a literature review on the damage-associated molecular patterns (DAMPs), receptors for advanced glycation end products (RAGE), negative soluble isoforms of RAGE, chemokine-stromal cell-derived factor-1 (SDF-1) and one of the adipokines, resistin, in the pathogenesis of preterm labour. We conclude that the chronic inflammatory response can play a much more important role in the pathogenesis of preterm delivery than the acute one.

## 1. Introduction

Preterm labour, defined as a birth taking place between 22nd and 37th weeks of gestation, affects 10 to 12% of all pregnant women. It is the most common cause of morbidity and mortality of newborns, as well in the United States as in Europe. It is estimated that about 30–35% of all premature labours proceed as iatrogenic, with maternal or fetal indications, while 40–65% complete spontaneously in the consequence of preterm uterine contractility or membrane rupture, or both [1]. Despite the rapid development of perinatal medicine over the past 20 years, constant increase can still be observed in preterm labour prevalence [2]. There are numerous risk factors of this pregnancy complication, which include not only infections, but also socioeconomic, demographic, environmental, and genetic influences. Anyway, it is highly probable that all of the above activate maternofetal inflammatory responses leading to uterine activity or preterm premature membrane rupture (pPROM) [3]. Many investigators believe preterm labour to be an acute obstetric disease related to ascending bacterial infection of the lower pole of conceptus with exogenic or endogenic microorganisms, with subsequent rapid maternal and fetal immunologic response [4–8]. The occurrence of increased levels of some inflammatory mediators not only in maternal blood and cervicovaginal discharge but also in amniotic fluid and placental or membrane samples is already well-known. In women diagnosed with chorioamnionitis and premature labour, in comparison to those who delivered at term, higher levels of interleukin 1 beta (IL-1*β*), calcium binding protein A5 (S100A5), prolyl 4-hydroxylase alpha polypeptide 2 (P4HA2), interleukin 6 (IL-6), interleukin 8 (IL-8), lipopolysaccharides (LPS), tumor necrosis factor alpha (TNF-*α*), and C-reactive protein (CRP) were found in maternal serum, cervicovaginal discharge, and amniotic fluid [9–12]. Unfortunately, increased levels of the aforementioned mediators can be detected only in case of overt or subclinical intrauterine infection what limits their usefulness, because the average time-to-delivery in most women presenting a high level of inflammatory cytokines does not exceed 7 days, which leads to the conclusion that in such cases there is no effective treatment option available.

## 2. Pathogenesis of Preterm Labour

Despite numerous studies on the aetiology and pathogenesis of preterm labour, its very cause still remains unclear. The activation of maternofetal immunologic response cascade is believed to be a causative agent in most cases. It is probable that activation of both chronic and acute inflammatory response patterns is necessary to result in premature birth. The microbes classified as physiologic and pathologic for women's vagina environment, including bacteria, fungi, and viruses, as well as their components such as lipopolysaccharides, heat shock proteins, and peptidoglycans, induce increased production of inflammatory cytokines via activation of toll-like receptors (TLR), especially the TLR-2 and TLR-4 type. The increased release of cytokines, mostly IL-1*β*, IL-6, IL-8, and TNF-*α*, leads to activation of arachidonic acid cascade, resulting in intensive production of prostaglandins and different types of proteases, especially metalloproteinases (MMPs). The prostaglandins are responsible for the onset of uterine contractility and cervical maturation, while MMPs are the main causative agent of membrane destruction.

What is noticeable, in most situations of developing infection located in proximity of conceptus, that is, the vagina or cervix, preterm delivery does not occur. Today, the presence of an additional predisposing factor is supposed to be necessary to make cytokines, prostaglandins, and proteases influence the effect on pregnancy complication. Such an additional factor is presumably chronic inflammation with its special mediators, so-called alarmines, whose intracellular form is known under the name damage-associated molecular patterns (DAMPs). Stimulated DAMPs interact via the receptor for advanced glycation end products (RAGE). There is a reasonable suspicion that the negative role of alarmines in preterm labour development is much more important than the influence of cytokines themselves, because of release of the latter only in the acute phase of inflammation.

The alarmines are most likely to cause chronic inflammation via activation of the TLR and RAGE. The chronic alarmines-mediated inflammation, with stimulation of RAGE and TLR should be assumed as a cause of preterm cervical maturation, meant as cervical effacement and its consistence change, in women who are going to develop premature labour. Contemporary sonographic evaluation of the cervix and estimation of cervicovaginal discharge for fetal fibronectin concentration are appreciated and widely used in clinical practice as preterm labour risk factors [13–15].

The pregnant woman, whose cervix length in sonographic evaluation does not reach 25 mm and who meets with a positive result of the fibronectin test, in most cases gives premature birth. This observation is well-proven, particularly in women who demonstrate clinical signs of threatening preterm labour, which leads to the conclusion that the diagnostic methods mentioned above cannot serve as a screening test to extract a group of those women who definitely need preterm labour prophylaxis. So far, the optimal method, allowing diagnosing accurately the asymptomatic women at risk of preterm birth, has not been invented. It appears that biochemical markers of chronic inflammation can fulfill the criteria. What should be emphasized is that the detection of increased levels of inflammatory cytokines is closely associated with subsequent delivery, occurring in a short time from the aforementioned markers manifestation. It is mostly related with the presence of intra-amniotic infection and with the lack of effective treatment options for preterm labour. Intensive tocolytic treatment applied in women who demonstrate increased levels of inflammatory cytokines significantly worsens neonatal prognosis, not extending the duration of pregnancy in reality. Antibiotic agents administration elongates the period of latency, that is, the interval from preterm membrane rupture to the spontaneous onset of uterine contractions, not improving neonatal prognosis. The research on the importance of inflammatory cytokines for diagnostics of preterm labour conducted between 1995 and 2008 proved clearly their function as the initiators of uterine contractility, premature preterm membrane rupture, and development of chorioamnionitis. But still none of them could be entitled as an early marker of threatening preterm labour, nor was used as a standard in the clinical practice. Today it is believed that high levels of all inflammatory cytokines are detectable too late to be an adequate indicator of the need for preterm labour treatment. For this reason most of the research concerns the analysis of the influence of chronic inflammation markers on the onset of premature labour [12, 16–20].

## 3. Chronic Inflammation as an Element of the Pathogenesis of Premature Labour

The inflammation is one of the body defense mechanisms, activated to control homeostasis in case of its disturbance caused by endogenic or exogenic factors [21]. Exogenic inflammatory factors, including bacteria, viruses, lipopolysaccharides, peptidoglycans, and viral RNA, generally named pathogen-associated molecular patterns (PAPMs), have their function of high importance in preterm delivery pathogenesis, associated mostly with intrauterine infection, which today is mostly well-known [22, 23].

In 2007 Oppenheim et al. suggested introducing the concept of alarmines to distinguish clearly the inflammation as an effect of exogenic influences from the inflammation as a response of the immune system caused by endogenic factors. Intracellular alarmines, also known as damage-associated molecular patterns (DAMPs), can moderate immune response as well via receptors TLR and RAGE as by direct activation of cytokines production in neutrophils or macrophages [24].

The best known alarmines include high-mobility group box-1 (HMGB1), heat shock proteins (HSP, mainly HSP 70), calcium binding protein (S100), hepatoma-derived growth factor (HDGF), interleukin 1-*α*, and the uric acid [25, 26].

The HMGB1, considered to be a prototypical alarmine, is a nuclear protein which can be released by most cells in case of their damage [27]. As is shown in Table 1, after its secretion from the cell, HMGB1 is able to cause both inflammatory response and tissue regeneration.

Most alarmines act via activation of nonspecific receptors, including TLR and RAGE. Activating nuclear factor kappa-B (NF-*κ*B), alarmines can stimulate cytokines and chemokines production and thereby initiate the inflammatory cascade.

There are very few studies on the importance of HMGB1 for preterm labour. Romero et al. detected its increased levels in the amniotic fluid in the pregnancies with premature labour and with intrauterine infection in both, regardless of whether the participants suffered from premature preterm membrane rupture or not. They also found a higher concentration of HMGB1 in amniotic fluid in women who experienced premature preterm membrane rupture compared to those who did not, concluding that HMGB1 can be responsible for membrane perforation in premature pregnancies [28]. Dubicke et al. found a higher expression of HMGB1 in women in active phase of labour comparing to those without uterine activity, suggesting alarmine to be associated with cervical maturation and dilation in mature delivery. Contrary to Romero et al., they detected lower levels of this substance in the pregnancies with premature preterm membrane rupture than in women affected with threatening premature labour but with maintained continuity of the membranes [18].

There are some more studies on premature labour concerning heat shock proteins (HSP) [25, 29, 30]. This subgroup of proteins, such as HSP 60, HSP 70, HSP 72, and HSP 90, belong to intracellular cytoprotective molecules, acting as endogenic TLR and RAGE ligands. The heat shock proteins enhance production of NF-*κ*B via their interaction with TLR and RAGE, which stimulates cytokines cascade, resulting in an increase in production of prostaglandins, having their effect on the uterine muscle and the cervical connective tissue. The HSPs also take their part in antigen presentation as well as in macrophages and lymphocytes activation [29]. Chaiworapongsa et al. studied heath shock protein-70 amniotic fluid concentration in women giving birth at term, in those suffering from threatening preterm labour and premature preterm membrane rupture and those presenting signs and symptoms of intrauterine infection. They found increased levels of HSP 70 only in giving birth at term participants and in the subgroup of women who met the criteria for confirming the intrauterine infection [25]. Fukushima et al. recognized HSP 70 as detectable in the pregnant women's serum in every trimester, reaffirming increased concentration of this molecule in participants being at risk of preterm labour in whom tocolytic treatment failed. They postulated the HSP 70 level in women with threatening premature labour to be a potential marker of tocolytic treatment effectiveness [30]. Having regard for the literature on the issue, it is clear that the alarmines have an important role in premature labour pathogenesis. Unfortunately, the majority of research proves a high concentration of the most commonly studied DAMP only in women suffering from threatening preterm birth accompanied by intrauterine infection. In the pregnancies at risk of premature labour but lacking the criteria of exogenic infection, increased levels of neither HMGB1 nor HSP were found.

Present studies on premature labour pathogenesis usually focus on the receptors for alarmines. The receptors for advanced glycation end products (RAGE) belong to the group of transmembrane multiligand receptors belonging to the immunoglobulin super family, activation of which is crucial, inter alia, for induction and maintenance of inflammatory response [31–33]. As shown in Table 2, the RAGE is localized on the surface of many cell populations, including phagocytes, hepatocytes, endothelium, smooth muscles of blood vessel media, nervous system cells, and mesangial cells of the glomeruli [34]. The RAGE determining gene is localized on the 6th chromosome, next to the major histocompatibility complex class III region [35]. This location makes RAGE probable not only to act as a receptor, but also to be involved in the reaction to different types of injury [36]. It has been proven that the RAGE gene expression can be induced in response to enhanced cell activation caused by increased concentration of RAGE ligands in case of tissue damage and inflammation [37]. Apart from the native RAGE form, there are some additional, mostly negative, isoforms described in the literature. The membrane form of the receptor, known as dominating negative RAGE (dnRAGE) can be found on the cell surface. The ligands attached to the receptor are concentrated on the cell surface, which results in suppression of the receptor's signal transduction [38, 39]. The soluble, negative, detectable in circulating blood, secretory variants of RAGE are known as sRAGE. The sRAGE-ligand complexes lose their affinity for heparin sulfate to be released to the blood stream and, eventually, captured and degraded in the liver or spleen. A particular type of sRAGE lacking the transmembrane and cytosolic domains, is thought to be alternatively spliced and named endogenous secretory RAGE (esRAGE) [40]. This heterogenic group of proteins, capable of binding, inter alia, advanced glycation end products (AGE), has a protective effect on blood vessels against toxic influence of ligand-RAGE complexes [41]. The ligand-RAGE interaction enhances oxidative stress, activating not only NADPH oxidase, but also some transcription activating factors, mainly NF-*κ*B and mitogen-activated protein kinase (MAPK) [42]. The active form of NF-*κ*B, after its translocation to the nucleus, activates the expression of genes of such cytokines as IL-1, IL-6, and TNF-*α*, and adhesion molecules like VCAM-1 (vascular cellular) and ICAM-1 (intracellular) participating in inflammatory response pattern [42, 43].

Formerly, the advanced glycation end products were believed to be the only RAGE ligand. Today it is known that RAGE is capable of binding most of the alarmines resulting in endogenic inflammatory cascade beginning. The RAGE-mediated inflammatory reaction can be moderated by RAGE negative forms, belonging as well to their transmembrane (dsRAGE), as to soluble (sRAGE, esRAGE) variations. It has been proven that high sRAGE concentration decreases systemic inflammatory response, thereby improving the natural history and the prognosis of some diseases linked to endogenous inflammatory processes. Such protective role of sRAGE was confirmed, for example, in cases of diabetes mellitus, in some circulatory system diseases, in a number of neoplasms, and, last but not least, in atherosclerosis [44–48].

The hypothesis of the protective role of RAGE negative variants yields the question, whether soluble RAGE concentration in pregnancies can influence the prevalence of premature labour connected to both spontaneous uterine contractility and preterm membrane rupture. Only a few authors took the issue of RAGE in premature labour, of which Romero is the leading investigator. In 2008 he evaluated sRAGE and esRAGE concentration in human amniotic fluid in the second trimester of pregnancy, at term in pregnancies without the signs of labour, at term in those in active phase of labour, in women suffering from premature labour having their fetal membrane intact, and in those affected with preterm premature membranes' rupture. He assessed receptors' concentration in the group of premature pregnancies depending on the presence or absence of intrauterine infection signs, concluding higher sRAGE levels to be linked to intra-amniotic contagion [49]. In women at their estimated date of delivery, showing signs of labour active phase, lower levels of RAGE were found, in contrast to those not giving birth. Knowing the molecular basics of RAGE significance for inflammatory response modulation, the results described above should be regarded as very surprising. In 2012 the same author published his scientific report on sRAGE concentration in the amniotic fluid depending on the presence or absence of chorioamnionitis. This time he found a decrease in the sRAGE level accompanying signs and symptoms of intra-amniotic infection [50]. Hájek et al. proved that sRAGE serum concentration is lower in pregnancies affected with pPROM comparing to those threatened with premature labour but with their membranes intact and that sRAGE level is higher in the group with threatening preterm birth than in healthy pregnant women [51]. Bastek et al. conducted a prospective analysis of sRAGE importance for premature labour aetiology, assessing sRAGE serum concentration in 529 women meeting the criteria for threatening preterm labour, of which 39.8% gave birth prematurely. In the latter group a significantly lower sRAGE level was found in contrast to participants who delivered at term. The authors concluded that sRAGE serum concentration can be useful as a prognostic marker of premature labour and that a high sRAGE level can announce a favourable prognosis. Another aim of the authors' analysis was the evaluation of umbilical cord blood sRAGE level in the neonates. They found lower prevalence of sepsis in the infants with high sRAGE cord blood concentration [17]. So far, the studies already conducted do not clearly prove the protective function of negative soluble RAGE isoforms in premature labour. It seems that there is a need for further research on this issue.

## 4. The Significance of Stromal Cell-Derived Factor-1 for Premature Labour Pathogenesis

The importance of the cytokines and acute inflammation in preterm labour aetiology is nowadays well-proven, while intensive research on the role of DAMPs and their receptors in this pregnancy complication is still in progress. It seems that, searching for causes of premature labour, one should also appreciate the significance of some chemokines. The chemokines are cytokines responsible for modulation of immune cells (i.e., leukocytes and lymphocytes) migration towards the area of inflammation. It is also known that they play an important role in neovascularisation, in cell and tissue regeneration, and in the modulation of inflammatory response to viral infections. There are four subfamilies of chemokines, determined according to their gene location. The *α*-chemokines gene lies in locus 4q12-21 on chromosome 4. The first two of four conservative cysteine residues of the *α*-chemokines are separated by a single amino acid, and because of it they are called CXC chemokines. The *β*-chemokines gene lies in locus 17q11-32 on chromosome 17. Their first two conservative cysteine residues lie in close proximity, which gives them the label of CC chemokines. The *γ*-chemokines, belonging to locus 1q23 on chromosome 1, contain only two of four conservative cysteine residues. They are classified as C chemokines. The *δ*-chemokines gene, lying on chromosome 16, is composed of three nonconservative amino acids between the first two cysteine residues, which yield the name of CX3C- or CXXXC-chemokines [52].

The SDF-1 is one of CXC-chemokines, produced by the bone marrow stromal cells and by the endothelium of the pancreas, spleen, ovaries, and the small intestine [53, 54]. Its biological effect needs a specific receptor CXCR4. The significance of SDF-1 CXCR4 for the obstetrics is yet poorly understood. There is an expression of SDF-1 in human trophoblast cells. It is probable that the activation of CXCR4 by SDF-1 is one of the sources of maternofetal immune tolerance, enabling normal development of the pregnancy. Presumably, it is SDF-1 what facilitates the trophoblast invasion into the endometrium and spiral arteries remodeling. It also enhances the VEGF expression, participating in neovascularisation processes in growing pregnancy [55].

There are very few studies on the potential relationship between SDF-1 and the complications of pregnancy. Tseng et al. conducted a prospective research to evaluate SDF-1 concentration in the amniotic fluid collected by amniocentesis in the second trimester of pregnancy. They found pregnancies in whom the SDF-1 levels are higher to be more prone to preterm delivery and their neonates to have low birth weight and to reach lower first minute Apgar scores [56]. Aminzadeh et al. analyzed the concentration of chemokines CXCL12 (SDF-1) and CXCL10 (IP-10) in umbilical vein serum and in women's serum after their preterm or normal delivery. They considered the concentration of SDF-1 in maternal serum in both subgroups to be comparable, whereas its cord blood level in the preterm neonates to be significantly higher. It was emphasized that the role of CXCL10 in premature labour can be more important than that of SDF-1, because the increased level of the first was observed in both, umbilical cord blood and maternal blood, in the circumstance of preterm birth [57]. So far, the role of SDF-1 in premature labour pathogenesis remains not clearly explained. The literature lacks reports that would assess the concentration of the cytokines described above depending on the meeting of clinical or laboratory criteria of intrauterine infection. There is also no data on potential contribution of SDF-1 in premature preterm membrane rupture.

## 5. The Role of Resistin in the Aetiology of Premature Labour

Resistin is an adipokine belonging to the family of cysteine-rich proteins called resistin e-like molecules (RELM) and produced mainly in peripheral blood inflammatory cells, monocytes, and macrophages [58]. It is also detectable in the cells of lungs, pancreatic beta islets, bone marrow, and placenta/trophoblast. It is proven that resistin gene expression can be modulated not only by glucocorticosteroids, thyroid hormones, growth hormone, and insulin, but also by the elements of different inflammatory signal patterns, such as NF-*κ*B and cytokines. No specific resistin receptor was isolated so far, which results in lacking information on intracellular patterns of resistin mechanism of action. The significance of resistin function in inflammatory response makes this molecule interesting especially in the context of pathogenesis of premature labour. Some cytokines, such as IL-6, TNF-*α*, or LPS, stimulate resistin expression. In vitro, resistin itself enhances the expression of VCAM and ICAM and activates endothelial production of endothelin-1. The CRP level and the white blood cell count are related to resistin concentration in the patients suffering from renal insufficiency [59]. The role of resistin in some inflammatory diseases was proved. Increased concentration of this molecule was found not only in synovial fluid in patients diagnosed with rheumatoid arthritis, but also in serum in those with inflammatory bowel diseases such as Crohn disease or ulcerative colitis [60].

There are very few studies on resistin action in premature labour. In 2009 Kusanovic et al. analysed amniotic fluid resistin levels in 648 pregnant women in the second and third trimester, as in postdelivery period. They found resistin to be present in amniotic fluid in both second and third trimesters and its concentration to increase with pregnancy growth. In women threatened with premature labour, presenting signs and symptoms of intrauterine infection, the level of resistin was higher with no difference for broken or intact fetal membranes. The onset of labour did not change amniotic fluid resistin concentration in mature pregnancy. The authors concluded that resistin can play an important role in the initiation of maternal and fetal inflammatory response to intrauterine infection [61]. The neonatal prognosis depends not only on the newborn's birth weight and gestational age, but also on the presence or absence of the complications resulting from congenital infection. Gursoy et al. measured serum resistin levels in 118 premature neonates, taking samples two hours after delivery. They found higher concentration of resistin in the neonates born after pPROM, compared to those with the membranes intact until delivery. The neonates, whose mothers were administered with glucocorticosteroids in prenatal period, presented significantly lower levels of resistin. A correlation between resistin level and IL-6 and CRP concentration was proved only in the newborns whose mothers were not given glucocorticosteroids. The author's conclusion was that prenatal glucocorticosteroids administration diminishes inflammatory response in the neonate, which is an important favorable prognostic factor [62]. Mazaki-Tovi et al. noticed increased resistin levels in the pregnancies suffering from acute pyelonephritis, correlating with inflammation intensity. They emphasized that resistin concentration can be an early marker of systemic inflammatory response syndrome (SIRS), which can lead to the iatrogenic preterm delivery [63].

## 6. Conclusion

There are randomized studies confirming the effectiveness of progesterone in prophylaxis of preterm labour in women with previous premature delivery [64, 65]. Such treatment is also effective in the pregnancies with poor sonographic cervical length in the second trimester [66]. In contrast, progesterone administration seems to be ineffective in reduction of the prevalence of premature deliveries in women presenting high blood cytokines concentration, because the increase of cytokines approaches not before intrauterine infection development [67]. It is certain that some women without a bad obstetric history, having normal cervical length in their second trimester and presenting cytokines level within the reference range, are going to experience preterm delivery anyway. There is a need to search for biochemical markers of premature labour to become able to preselect a subgroup of the pregnancies as described above as potential beneficiaries of progesterone treatment. It seems that such novel markers are more probable to be found among DAMPs, chemokines, adipokines, or heat shock proteins, than among classic membrane phospholipids degradation products.

## Figures and Tables

**Table 1:** Characteristics of the alarmines important for premature labor pathogenesis.

Alarmine	Receptor	Action
HMGB1	TLR (toll-like receptor) RAGE (receptor for advanced glycation end products)	Inflammatory response (NF-*κ*B activation) Tissue regeneration

Heat shock proteins: HSP 60 HSP 70 HSP 72 HSP 90	TLR RAGE	Cytoprotection Antigen presentation Macrophage and lymphocyte activation Inflammatory response (NF-*κ*B activation)

**Table 2:** The RAGE variants characteristics.

Receptor	Location	Activation effect
Native RAGE	Cell surface: Phagocytes Hepatocytes Endothelium Smooth muscles of blood vessel media Nervous system cells Glomeruli mesangial cells	Induction and maintenance of inflammatory response

Dominating negative RAGE (dnRAGE)	Cell surface	Signal transduction suppression by concentration of ligands on cell surface

Soluble secretory RAGE (sRAGE) Endogenous secretory RAGE (esRAGE)	Ligand-RAGE complexes released to circulating blood, degraded in liver and spleen	Binding of advanced glycation end products
